# The Effect of Plate Thickness on Fusion, Complications, and Outcomes in Anterior Cervical Spine Surgery

**DOI:** 10.7759/cureus.41048

**Published:** 2023-06-27

**Authors:** Daniel Park, Anthony Arveschoug, Steve Wahlmeier, Graysen Petersen-Fitts, Phil Zakko

**Affiliations:** 1 Orthopaedic Surgery, Beaumont Health, Royal Oak, USA

**Keywords:** dysphagia, prevertebral soft tissue swelling, fusion rates, plate thickness, anterior cervical discectomy and fusion

## Abstract

Background and objective

Anterior cervical discectomy and fusion (ACDF) is a common surgery involving the cervical spine. The goals of ACDF include obtaining a solid fusion and minimizing complications such as dysphagia. The effect of plate thickness on fusion, dysphagia, and patient outcomes is not well established. In light of this, this study aimed to evaluate the effect of plate thickness on fusion rates, complications, and outcomes in ACDF.

Methods

A case-control study involving ACDF performed by a single surgeon was conducted with the aim of comparing two commercially available plating systems: the Medtronic plate (Atlantis Vision Elite, Medtronic, Memphis, TN) and Aegis plate (CastleLoc-P, Aegis, Englewood, CO). The patients treated with the Medtronic plate served as the control group (Std) as the plate is widely utilized, while those treated with the Aegis plate, which is touted as one of the thinnest plates on the market, constituted the case low-profile group (LP). Demographic variables, fusion status, and patient-reported outcome measures (PROM) were compared between the two systems.

Results

Baseline demographic data were not significantly different between groups. The LP plate group had a significantly lower rate of fusion per patient as well as per level at 12 months. PROM did not significantly differ at any time point between the groups. Dysphagia scores could be correlated with radiographic measures reported in the Prevertebral Soft Tissue Swelling - Index (PVSTS-I).

Conclusion

Based on our findings, the plate thickness was not associated with dysphagia rates; however, the use of a thinner plate correlated with a lower rate of radiographic fusion at 12 months. The PVSTS-I may be useful for identifying patients with abnormal and severely abnormal dysphagia scores.

## Introduction

Anterior cervical discectomy and fusion (ACDF) and anterior cervical corpectomy and fusion (ACCF) are increasingly common surgeries of the cervical spine [1]. Anterior cervical fusion is typically a highly successful surgery associated with a high percentage of good clinical outcomes [2]. Over 90,000 ACDF patients were identified between 2006 and 2010, making it the most commonly performed cervical spine procedure in the United States [3]. The goals of ACDF include decompressing neural elements, obtaining a solid fusion, and minimizing complications [4].

Since its adoption in clinical practice, ACDF has evolved to include anterior cervical plate constructs. The purpose of these anterior plate constructs is to foster immediate stability, maintain cervical lordosis, promote solid bony fusion, and prevent cage subsidence [5]. A non-plated approach is less invasive and does not require vertebral screw fixation; however, it has a higher rate of cage subsidence and cervical pseudarthrosis [6]. Despite the advantages offered by anterior plating, the technique is associated with plate-related complications such as postoperative dysphagia, screw breakage or pullout, and screw-plate migration [7].

Oropharyngeal complications following ACDF such as dysphagia, hoarseness, or esophageal perforation are often discussed in the literature [8]. The incidence of dysphagia within one week after ACDF varies from 1% to 79% depending on the definition used [9,10]. Large anterior cervical osteophytes have been shown to cause preoperative dysphagia due to mechanical disruption [11]. In addition, many studies have investigated the relationship between prevertebral soft tissue swelling (PVSTS) and postoperative dysphagia [8,12].

There are many commercially available anterior plating systems that can be used when performing ACDF. Plate thickness has been studied as it relates to postoperative dysphagia rates [13]. Plate thickness varies considerably among various anterior plating systems. The purpose of this study was to investigate the impact of plate thickness on patient outcomes following ACDF, especially postoperative dysphagia, by utilizing a standardized dysphagia scoring system.

## Materials and methods

The study was conducted in accordance with the Institutional Review Board (IRB) guidelines and the protocol was approved prior to initiation of the study. We adhered to all local, state, and federal regulations. After obtaining the IRB approval, we employed a retrospective case-control design to evaluate the procedures conducted by a single surgeon. Two anterior cervical plate systems were compared: the Medtronic plate (Atlantis Vision Elite, Medtronic, Memphis, TN) and the Aegis plate (CastleLoc-P, Aegis, Englewood, CO). The patients treated with the Medtronic plate served as the control group (Std) as the plate is widely utilized, while those treated with the Aegis plate, which is touted as one of the thinnest plates in the market, constituted the case low-profile group (LP). The Std plate has a thickness of 2.5 mm, and the LP has a maximum thickness of 2.0 mm. The data of all patients who underwent anterior cervical spine surgery utilizing one of these devices between February 2017 and December 2018 were collected for review. The inclusion criteria were age >18 years and anterior cervical procedure with one of the two listed implants. The exclusion criteria were primary diagnosis of trauma, tumor, or infection. Patients who received the thinner implant were considered the case group (Aegis, n=22), and the control group comprised patients who received the thicker plate (Medtronic, n=48).

Surgical procedure

A senior orthopedic spine surgeon (DKP) performed all surgeries utilizing a standard left-sided Smith-Robinson approach. Discectomies were performed utilizing a combination of pituitaries, Kerrison rongeurs, curettes, and a high-speed burr. PEEK cages packed with local autograft bone mixed with demineralized bone matrix were used as graft material. Aegis PEEK cages were paired with the Aegis plate and Medtronic cages were paired with Medtronic plates. Anterior cervical plates with variable angle screws were used following graft placement (Aegis/Medtronic). The only significant difference in operative or postoperative procedure between the case and control group lay in the anterior cervical plate and cage utilized.

Data collection

Baseline demographic variables (age, BMI, smoking status, revision status), as well as data relating to postoperative complications and reoperations, were recorded. Postop follow-up included lateral cervical radiographs, Neck Disability Index (NDI), Visual Analog Score (VAS)-Neck, VAS-Arm, and Eating Assessment Tool-10 (EAT-10) outcome scores at six weeks, three months, six months, and 12 months. Lateral flexion/extension radiographs were obtained at three months, six months, and 12 months to assess the fusion status. Radiographic fusion was determined based on the description by Cannada et al. with flowing bone through the interbody space and/or less than 2 mm motion between spinous processes on lateral flexion and extension views [14]. Radiographic assessment of PVSTS, as well as the Prevertebral Soft Tissue Swelling - Index (PVSTS-I), was determined based on the findings of Lee et al. [15]. Briefly, the soft tissue shadow was measured on a lateral radiograph anterior to the midpoint of vertebral bodies C3-C7. The width of the vertebral body was measured at the midpoint of each vertebral body. A ratio for each vertebral body was determined by dividing the width of the soft tissue shadow by the width of the vertebral body. The PVSTS-I is the average of the ratio for C3-C5.

Statistical analysis

Baseline demographic variables were compared between the groups. Continuous variables were compared using the student’s t-test. Nominal or ordinal variables were compared using a Z-test. Fusion status was assessed with Pearson's chi-squared test. All tests had statistical significance defined at p<0.05.

## Results

A total of 70 patients were included in the study: 22 in the LP group and 48 in the Std group. The difference between case and control groups was not statistically significant in terms of age (54.41 vs. 56.21, p=0.28), BMI (31.87 vs. 30.23, p=0.13), level of operation, number of levels operated on, adjacent level surgery (2 vs. 7, p=0.52), and smoking status (Table 1).

**Table 1:** Baseline demographic data Demographic characteristics of the standard thickness plate group (std) and low-profile plate group (LP) demonstrating no significant differences between the groups BMI: body mass index; SD: standard deviation

Demographic characteristics		LP	Std	P-value
Age, mean ± SD		54.41 ± 11.59	56.21 ± 11.76	0.28
BMI, mean ± SD		31.87 ± 5.96	30.23 ± 5.94	0.13
Level operated on, n				0.81
	C3/4	2	7	
	C4/5	8	21	
	C5/6	17	35	
	C6/7	13	20	
	C7/T1	1	3	
Number of levels				0.32
	1	5	18	
	2	15	23	
	3	2	6	
	4	0	1	
Adjacent level, n		2	7	0.52
Smoking status, n				0.66
	Current	5	10	
	Former	6	9	
	Never	11	29	

There was a significant difference between the groups with regard to radiographic fusion. The LP group had 68.2% of patients fused at 12 months compared with 93.8% of Std patients (p=0.0045). In addition, the LP group had 82.5% of attempted levels fused at 12 months compared with 96.6% of Std patients (p=0.0063) (Tables 2, 3).

**Table 2:** Fusion status per patient Comparison of fusion status per patient at 6 and 12 months between the standard thickness plate group (Std) and low-profile plate group (LP) revealing a significant difference in radiographic fusion at the 12-month time point favoring the standard plate

Study groups	N	6 months	12 months
LP, n (%)	22	13 (59.1%)	15 (68.2%)
Std, n (%)	48	36 (75.0%)	45 (93.8%)
P-value		0.18	0.0045

**Table 3:** Fusion status per level Comparison of fusion status per level at 6 and 12 months between the standard thickness plate group (Std) and low-profile plate group (LP) demonstrating a significant difference in level fusion percentage between the two groups favoring the standard plate

Study groups	N	6 months	12 months
LP, n (%)	40	31 (77.5%)	33 (82.5%)
Std, n (%)	87	71 (81.6%)	84 (96.6%)
P-value		0.59	0.0063

Patient-reported outcome measures (PROM) were obtained and compared for all time points between groups. The difference between the groups was statistically significant for VAS-Neck at 12 months with the LP group having a score of 1.10 vs. 2.84 for the Std group (p=0.04). At all other time points, the EAT-10, NDI, VAS-Neck, and VAS-Arm were not significantly different between the groups (Table 4). 

**Table 4:** Patient-reported outcome measures Comparison of patient-reported outcome measures between the standard thickness plate group (Std) and low-profile plate group (LP) demonstrating a difference in VAS-Neck scores at the 12-month time point favoring the low-profile plate EAT-10: Eating Assessment Tool-10; NDI: Neck Disability Index; SD: standard deviation; VAS-Neck/Arm: visual analog scale for neck and upper extremity

Outcome measures		LP, mean ± SD	Std, mean ± SD	P-value
EAT-10	Preop	0.95 ± 1.75	1.03 ± 3.12	0.46
	6 weeks	3.19 ± 4.21	3.77 ± 6.12	0.36
	3 months	3.50 ± 6.31	2.75 ± 4.58	0.32
	6 months	2.00 ± 4.01	1.86 ± 2.89	0.45
	12 months	0.75 ± 1.29	0.89 ± 2.03	0.41
NDI	Preop	49.00 ± 24.52	41.24 ± 16.30	0.10
	6 weeks	33.90 ± 23.89	36.80 ± 18.35	0.31
	3 months	31.40 ± 21.15	28.07 ± 18.45	0.28
	6 months	22.16 ± 20.20	22.14 ± 18.72	0.50
	12 months	18.58 ± 16.62	26.44 ± 23.40	0.14
VAS-Neck	Preop	4.68 ± 2.51	5.46 ± 2.82	0.16
	6 weeks	2.11 ± 2.26	2.42 ± 2.32	0.32
	3 months	1.74 ± 2.32	2.25 ± 2.51	0.24
	6 months	1.78 ± 2.52	2.34 ± 2.74	0.24
	12 months	1.10 ± 1.62	2.84 ± 3.15	0.04
VAS-Arm	Preop	4.92 ± 2.98	4.13 ± 2.76	0.17
	6 weeks	2.68 ± 3.24	1.93 ± 2.43	0.18
	3 months	1.75 ± 2.40	1.53 ± 2.14	0.37
	6 months	1.93 ± 2.93	2.49 ± 3.14	0.27
	12 months	2.68 ± 6.29	2.49 ± 3.30	0.45

A secondary purpose of this study was to determine if PROM dysphagia scores (EAT-10) would correlate with radiographic findings. In both the LP and Std groups, patients with an abnormal EAT-10 score had a significantly higher PVSTS-I compared to those with a normal EAT-10 score: LP group: 0.573 vs 0.633, p=0.036; Std group: 0.510 vs. 0.667, p<0.00001. Furthermore, the difference was more pronounced with patients who had a “severe” EAT-10 score in both the case and control groups: LP group: 0.573 vs. 0.735, p=0.044; Std group: 0.510 vs. 0.846, p=0.000028 (Table 5).

**Table 5:** EAT-10 score vs. PVSTS Comparison of patients reporting normal, abnormal, and severe EAT-10 scores with PVSTS measurements for the standard thickness plate group (Std) and low-profile plate group (LP). Patients treated with standard-profile plates demonstrated increased PVSTS measurements among those reporting abnormal or severe EAT-10 scores EAT-10: Eating Assessment Tool-10; PVSTS: prevertebral soft tissue swelling: SD: standard deviation

Study groups	Normal EAT-10, mean ± SD	Abnormal EAT-10, mean ± SD	Severe EAT-10, mean ± SD	Normal vs. abnormal	Normal vs. severe
LP	0.573 ± 0.132	0.633 ± 0.152	0.735 ± 0.080	P=0.036	P=0.044
Std	0.510 ± 0.136	0.667 ± 0.182	0.846 ± 0.279	P<0.00001	P=0.000028

## Discussion

The results of our study indicate that there is no statistically significant association between plate thickness and rates of dysphagia following ACDF; however, the use of thinner plates was associated with decreased fusion rates at 12 months. These results suggest that a construct with a thinner plate may not provide adequate stability for fusion when compared to a thicker plate construct.

Complications following cervical spine procedures, such as pseudoarthrosis requiring reoperation, are well described in the literature [7,16]. While reports on the impact of pseudoarthrosis on clinical outcomes are somewhat conflicting, studies generally suggest that successful bony fusion significantly increases the likelihood of favorable results after ACDF [17,18]. Concerns over the need for revision surgery may impact both the choice of index operation and the choice of implant system used. Several studies have compared anterior cervical plating systems with zero-profile implants or standalone devices for ACDF [19,20]. However, there is a scarcity of data in the literature when it comes to directly comparing PROM and radiographic outcomes of two anterior plating systems with different plate thicknesses. Our results suggest that a lower-profile implant system may not provide adequate stability to encourage reliable fusion.

Our study also indicated that decreased plate thickness was not associated with any significant improvement in PROM including dysphagia rates. The only statistically significant difference noted was that in the VAS-Neck at 12 months; this difference, however, did not reach a validated minimal clinically important difference (MCID) [21]. Instead, dysphagia scores correlated with PVST-I, which is more likely due to prevertebral swelling from surgical insult rather than plate thickness. This is somewhat surprising and contrasts with some existing literature. It is well known that large anterior osteophytes can cause dysphagia; thus, it seems intuitive that thicker anterior cervical plates may also cause dysphagia [11,22]. In addition, a study by Lee et al. [23] that examined dysphagia rates following ACDF in patients who received implants of different contours and a difference in thicknesses of 0.9 mm found a statistically significant difference in dysphagia rates between the two plates, suggesting the thicker plate predisposed patients to dysphagia.

Our study failed to replicate any such difference in dysphagia rate based solely on plate thickness. Our findings were more in line with those of Chin et al. [13] who also did not find any correlation between plate thickness and dysphagia rates postoperatively. Our study improves upon their findings by utilizing a standardized dysphagia scoring system. It is important to note that many predisposing factors for dysphagia have been documented in the literature. Estimated blood loss, length of surgery, gender, number of levels fused, and highest cervical level containing a plate have all been studied as potential causes of dysphagia [24]. While our baseline patient demographic data showed no statistically significant differences and all surgeries were performed by the same senior orthopedic spine surgeon, it is possible that there was variability in some of these predisposing factors, which may have contributed to differences in dysphagia rates.

This study can be criticized for a lack of direct evidence that the plates used in the study led to pseudarthrosis or the need for reoperation. Furthermore, two different brands of plates and cage constructs were compared, and the results may have been influenced by differences in the interbody devices. Additionally, our small sample size was relatively small, with only 22 patients receiving the LP implant. Finally, we studied these patients and their outcomes in a retrospective fashion without randomization. Hence, it is difficult to draw any definitive conclusions about the choice of the implant in patients undergoing ACDF based on our data. However, this study does provide a solid foundation upon which further research investigating the relationship between anterior cervical plate thickness and patient outcomes may build.

## Conclusions

ACDF is a highly effective surgery to address a range of cervical spine pathologies; however, complications, including dysphagia and pseudoarthrosis, do occur and may be related to the fusion construct in some cases. The goal of this study was to investigate the impact of plate thickness on patient outcomes and fusion rates. We found that decreased plate thickness was not associated with increased PROM scores or decreased dysphagia rates. In addition, the low-profile plate was associated with increased reoperation rates and pseudoarthrosis. These results suggest no current clinical benefit to decreasing plate thickness. However, continued research is necessary, particularly given the rapidly evolving landscape of cervical fusion hardware.
